# 

**Published:** 1892-06-25

**Authors:** 


PRICE TWOPENCE.

No. 300, Vol. XII.—June 25th, 1892.

Registered at the General Post Office as a Newspaper for transmission in the United Kingdom and Abroad.]

WITH EXTRA NURSING SUPPLEMEN

Per Annum, 8s. 6d., post free.

Monthly Parts, 6d.; post free, 8d.

Ditto per Annum, 8s., post free.

